# Supplementary material for: A Dual-Acting Nitric Oxide Donor and Phosphodiesterase 5 Inhibitor Activates Autophagy in Primary Skin Fibroblasts
Source: Int J Mol Sci. 2022 Jun 20;23(12):6860. doi: 10.3390/ijms23126860 (PMC9224465; doi:10.3390/ijms23126860)
Supplement: Supplementary file 1 [file ijms-23-06860-s001.zip › ijms-1682541-supplementary figures.pdf]

A Dual-Acting Nitric Oxide Donor and Phosphodiesterase 5 Inhibitor Activates Autophagy in Primary Skin Fibroblasts

Esther Martínez-Martínez[1], Paola Atzei[2], Christine Vionnet[1], Carole Roubaty[1], Stephanie Kaeser-Pebernard[1], Reto Naef[2], and Jörn Dengjel[1]

[1] Department of Biology, University of Fribourg, 1700 Fribourg, Switzerland

[2] Topadur Pharma AG, Grabenstrasse 11A, 8952 Schlieren, Switzerland

Supplementary Figures

- **Supplementary Figure S1.** PCA of NHK.
- **Supplementary Figure S2.** Complete network analysis of significantly enriched proteins in TOP-N53 treated NHFs.
- **Supplementary Figure S3.** Analysis of mTORC1 signaling pathway and autophagy-related markers.

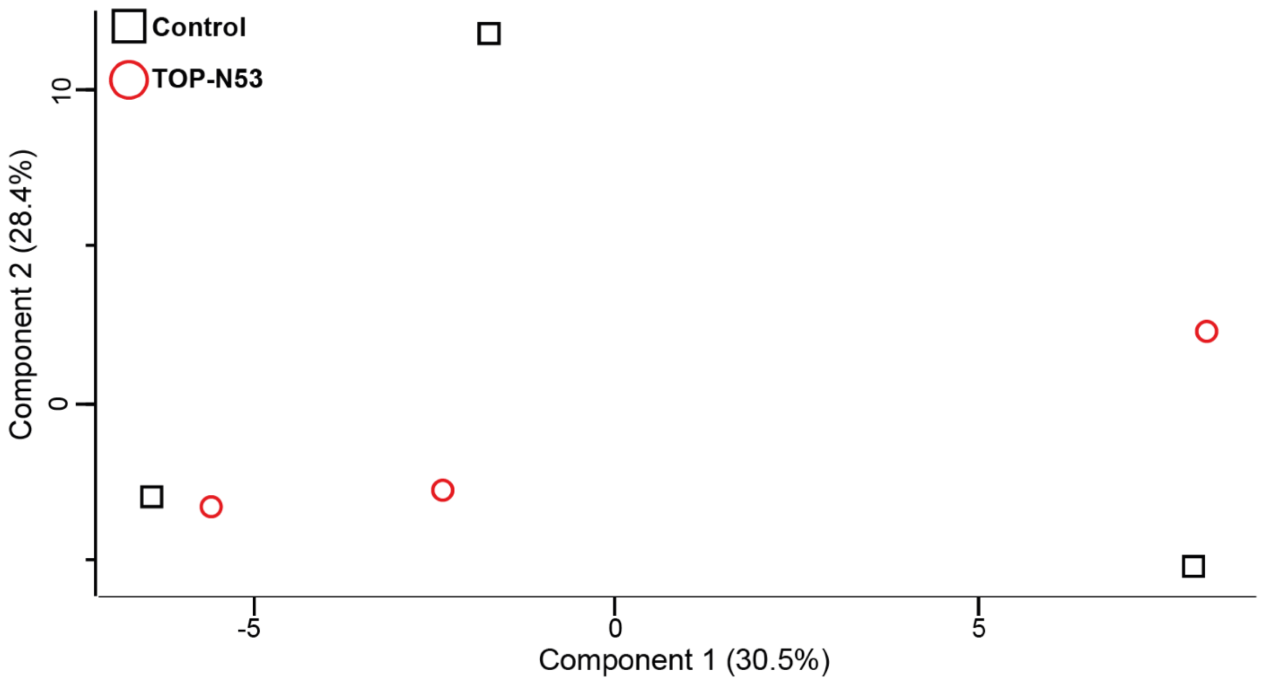

Supplementary Figure S1. PCA of NHK. Log$_2$-transformed LFQ intensities of identified proteins were used. The two experimental groups (Control and TOP-N53 treatment) do not separate, indicating no global effect of TOP-N53 on the proteome of NHK.

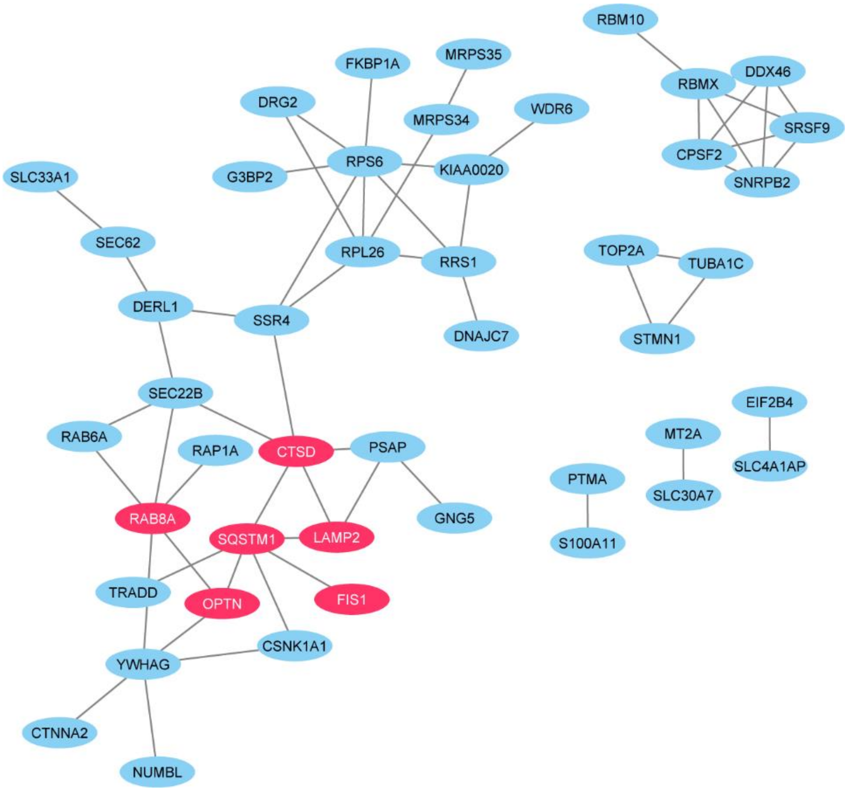

Supplementary Figure S2. Complete network analysis of significantly enriched proteins in TOP-N53 treated NHFs. Protein-protein interactions of 63 significantly enriched proteins in TOP-N53 treated NHF as analyzed by STRING DB (FDR < 0.05). Red labeled proteins carry terms "autophagy" (GOBP). Proteins that were not part of a node of at least 2 proteins were not included (n = 48).

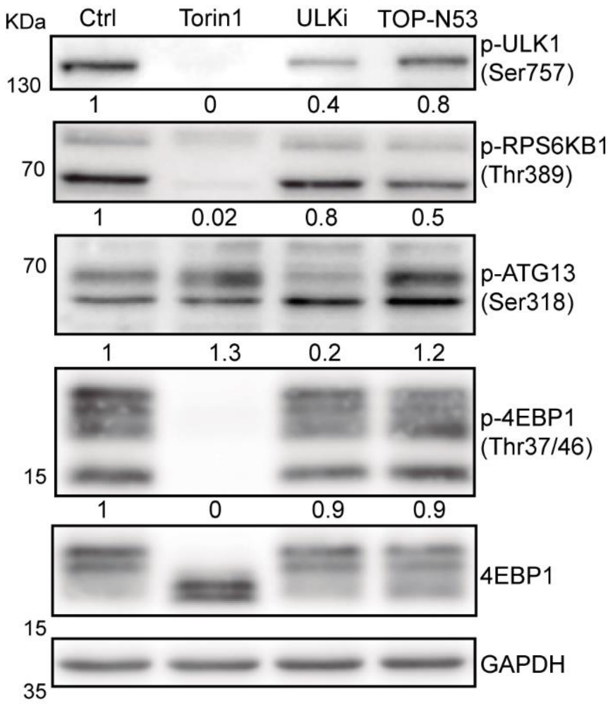

Supplementary Figure S3. Analysis of mTORC1 signaling pathway and autophagy-related markers. Analysis of different markers related to mTORC1 signaling and autophagy upon TOP-N53 treatment. NHFs were treated with TOP-N53 (1 □M, 24 h), the specific ULK1 inhibitor MRT68921 (1 □M, 2 h) and the mTORC1 inhibitor Torin-1 (1 □M, 2 h) for comparison of triggered signaling. Representative western blot (n = 2) and its quantification below each protein panel (normalized to GAPDH).
